# Echoes of childhood trauma: the relationship between adverse childhood experiences, brain structure, and mental health in aging adults

**DOI:** 10.1038/s41398-026-03811-2

**Published:** 2026-02-03

**Authors:** Anne Klimesch, Leonie Ascone, Götz Thomalla, Bastian Cheng, Marvin Petersen, Ingo Schäfer, Jürgen Gallinat, Simone Kühn

**Affiliations:** 1 Department of Psychiatry and Psychotherapy, University Medical Center Hamburg Eppendorf, Martinistraße 52, 20251 Hamburg, Germany; 2 Department of Neurology, University Medical Center Hamburg Eppendorf, Martinistraße 52, 20251 Hamburg, Germany; 3 Center for Environmental Neuroscience, Max Planck Institute for Human Development, Lentzeallee 94, 14195 Berlin, Germany

**Keywords:** Depression, Neuroscience

## Abstract

Adverse childhood experiences (ACEs) are associated with persistent mental health risks and brain structural differences across adulthood, yet their long-term neurobiological relevance in aging populations remains unclear. Given that ACEs are common and societies are aging, understanding how early adversity relates to mental and brain health across the lifespan is an important goal. This preregistered study examined whether ACEs are associated with mental health symptoms in mid- to late adulthood, and whether regional brain structure may account for part of this relationship. Cross-sectional data of the Hamburg City Health Study were used, involving participants aged 46–78 years with available magnetic resonance imaging data (N_total_ = 2 624; eligible: n_analysis sample_ = 1 900). Mental health status was quantified using the Patient Health Questionnaire-9 and the General Anxiety Disorder-7 questionnaire, while ACEs were assessed using the 10-item ACE-questionnaire. Predefined regions of interest (ROI) were the hippocampus, amygdala, and dorsolateral prefrontal cortex. Bayesian statistical analysis provided strong evidence for an association between ACEs and both mental health outcomes but did not confirm the hypothesized mediation by ROI-level brain morphology. Exploratory whole-brain voxel-based morphometry revealed significant regional grey matter volume (rGMV) reductions in individuals with 3 or more ACEs, affecting bilateral limbic and frontal regions—including the nucleus accumbens, gyrus rectus, and insula. These reductions were more pronounced and widespread in individuals with 4 or more ACEs, extending to areas in the dorsolateral and medial prefrontal cortex, anterior cingulate cortex, inferior parietal and temporal gyri, occipital cortex, and cerebellum. Notably, no increases in rGMV were observed for any ACE group. This suggests a dose-dependent effect, with 4 or more ACEs marking a potential threshold for more distributed neuroanatomical alterations. These results derived from a representative general population study extend the understanding of the possible effects of ACEs by providing evidence that structural changes associated with ACEs could last into mid and late adulthood similarly to mental health outcomes.

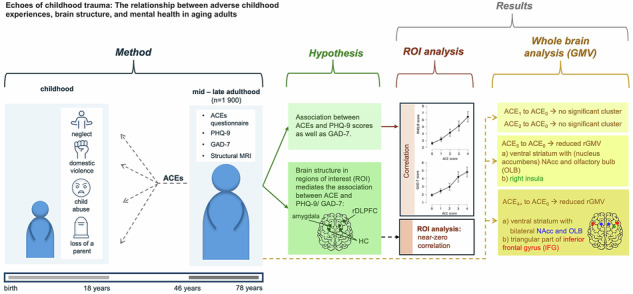

## Introduction

Disturbing events that occur during childhood or youth can have detrimental effects on life outcomes. Such potentially traumatic experiences that occur before the age of 18 years involve, among other distressing experiences, domestic violence, child abuse, or neglect, and are referred to as adverse childhood experiences (ACE) [1]. About 44% of individuals in a German sample (N = 2 531, >13 years of age) reported having experienced at least one ACE and about 9% reported multiple ACEs [2]. Even more than 60% of ageing individuals (45–85 years) in a Canadian study reported at least one ACE (N = 44 817) [3]. ACEs are contextual, being associated with individual circumstances as the socioeconomic status (SES) [4]. However, we are facing increasing global threats, e.g., the Covid-19 pandemic, wars and the climate crisis, which disproportionately affect vulnerable groups. Global threats thus contribute to ACEs and their compound effect may increase health disparities [5]. In the long run, ACEs are a risk factor for adult mental health disorders [2], physical conditions [1, 6], as well as deviations in brain structure that persist into adulthood [7]. At the same time, the global population is ageing which implies a need to understand the mechanisms underlying the long-term effects of ACEs and how these may increase health disparities.

Mental health outcomes of ACEs can be manifold. For example, in early adulthood, individuals with ACEs are more likely to show maladaptive cognitions regarding themselves and social relationships [7, 8]. This can comprise the profound expectation that people cannot be trusted. In line with this, ACEs show a graded relationship with poor quality of life [6], as well as with psychological distress [1] in adulthood. Beyond general mental health outcomes, ACEs are associated with mental disorders such as major depression [5, 8] general anxiety disorder [9], and even with increased suicide attempts [6].

In addition to mental health outcomes, past research has revealed a relationship between ACEs and regional brain volume in adulthood: hippocampal and right dorsolateral prefrontal cortex (rDLPFC) volumes were reduced in individuals with and without a diagnosis of a mental health disorder and independent of the type of ACE [10]. Volume deviations are largest in individuals with mood disorders compared to healthy samples [10]. In contrast, deviations in amygdala volume are only observed in age subgroups: in healthy adolescents (12–17 years), ACEs are associated with bilaterally reduced amygdala volume [11], but in a meta-analysis including adult samples this was only the case for individuals diagnosed with a mental disorder [10]. Thus, hippocampus and rDLPFC appear to decrease in volume following ACEs, but amygdala volume might normalize over time in individuals who do not develop a mental disorder. Importantly, while most of past studies have been performed in young and middle-aged samples with a mean age of 21–41 years, Gerritsen et al. [7] have investigated an older sample (60–96 years) and found reduced hippocampal and amygdala volumes with increasing age only for those with at least two ACEs. Thus, there is increasing evidence that age plays an important role in the volumetric trajectories of the rDLPFC, and hippocampal and amygdala volume following ACEs.

Right DLPFC, hippocampus, and amygdala also show anomalies in mood disorders and it has even been suggested that their structure can distinguish between unipolar and bipolar depression [12] as well as contribute to neural diagnostic markers for further disorders [13]. Regional brain volume reductions have been proposed as an ACE-induced risk factor for depression and anxiety, as individuals with these disorders differ on hippocampus volume depending on the presence of ACEs [14]. The question arises how exactly ACEs, regional brain volume deviations, and mental disorders relate to each other in adulthood. Different hypotheses have evolved: Rao et al. [15] found a partially mediating effect of brain volume on the relationship between ACEs and depression while Paquola et al. [10] have identified mental health as a moderator of the relationship between ACEs and brain volume during adulthood.

The current state of research on ACEs and adult outcomes clearly indicates that ACEs have long-term detrimental effects at both the symptom as well as neural levels. However, samples of past studies predominantly included individuals between 18 and 60 years of age, thus, little is known about the effects of ACEs in later adulthood. In addition, results are still inconclusive regarding the role of volumetric deviations on the relationship between ACEs and adult mental disorders. Importantly, there is one study that analysed a large dataset on ACE (N = 6 751) using data of the UK Biobank [16], however, other samples of past research that involve MRI data included N = 14 to N = 860 individuals [17, 18]. In a meta-analysis of 38 studies only six had a sample size above N = 100 [10]. This displays a critical limitation to past studies which is addressed here. To our knowledge, no previous study has tested for a potential mediating effect of regional brain volume on the relationship between ACEs and mental disorders in the ageing general population.

The aim of this study was, first, to identify if previously reported associations between ACE and brain structure as well as mental health are observable in mid to late adulthood. We investigated the following pre-registered research question: what is the relationship between ACE and regional brain structure in their effect on mental health in mid to late adulthood? We hypothesized that rDLPFC thickness, as well as amygdala and hippocampal volume mediate the association between childhood adversity and mental health in mid to old age while controlling for sex, total intracranial volume (TIV), and SES. Further, we expected that age moderates the association between ACE and thickness/ volume in the regions of interest (ROIs). To test these hypotheses, we utilized data from the observational Hamburg City Health Study, a large cohort study which aims to identify somatic and psychological risk or resilience factors for common somatic and mental disorders in the ageing general population of Hamburg, Germany [19].

## Materials and methods

**Fig. 1:**
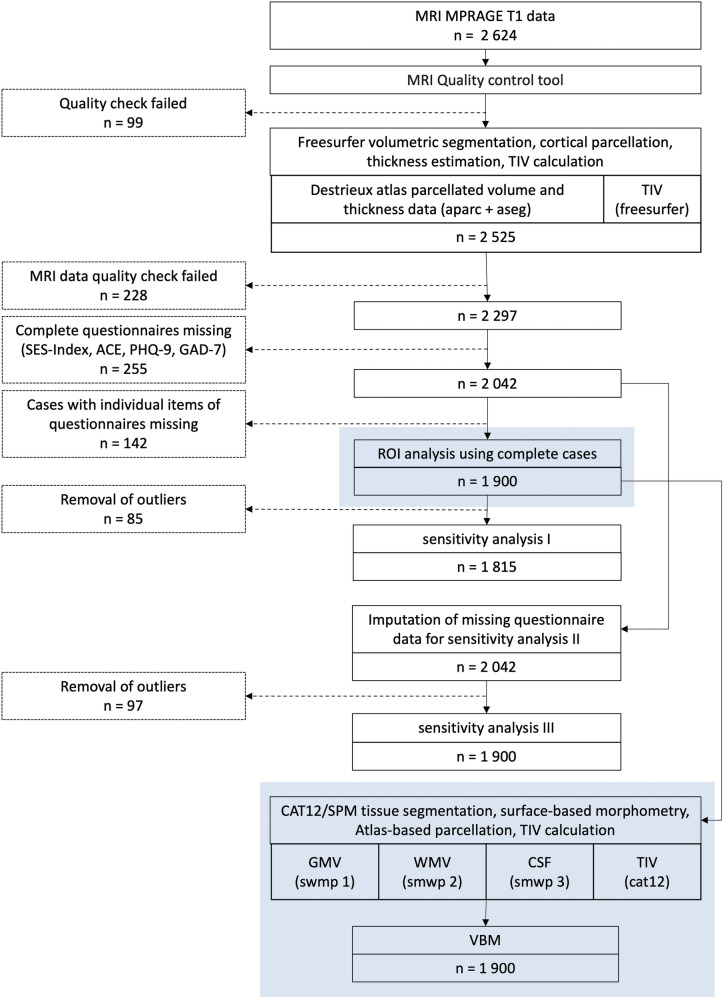
Flow-chart outlining the data in-/ exclusion process for the final analysis sample. The pre-registered ROI-hypotheses were tested in the original sample constituting n = 2 624. During the ROI-pre-processing n = 327 participants were excluded due to missing data or low MRI-data quality and n = 397 participants were excluded due to missing data on the behavioural variables. The sample used in the ROI-hypotheses n = 1 900 was subsequently also used in the exploratory VBM analyses to assure comparability (both coloured in blue).

The study was pre-registered with Open Science Framework (10.17605/OSF.IO/ZE6BD, registration date: 14/04/2023). We used cross-sectional first-wave data from the Hamburg City Health cohort-study (N = 2 624), which involved participants between 46 and 78 years of age with eligible magnetic resonance imaging (MRI) data, randomly sampled from the residents’ register of Hamburg stratified by sex and age (data collection: 2016–2022). Importantly, this sample constitutes only those participants of the cohort study, who chose to participate in the MRI scan and provided suitable data (n = 7 479 participants opted out; reasons are unknown). Detailed information on the data collection process can be found in the paper describing the study design of the Hamburg City Health Study [19]. Figure 1 displays a flow-chart outlining the exclusion process and the final sample size.

### Measures

#### Questionnaire data

We used the authorised German version of the ACE questionnaire [20, 21] to operationalize childhood adversity. The questionnaire comprises 10 items assessing different types of potentially traumatic experiences before the age of 18 years (full questionnaire in Supplementary Table 1). Five of these items address five different types of potentially traumatic experiences: emotional abuse, emotional neglect, physical abuse, physical neglect, and sexual abuse, and five items assess household dysfunction [1]. The questionnaire has a binary response format identifying whether an individual reports to have made the respective childhood experience. The sum score ranges from 0 (no ACEs) to 10 (maximum possible number of ACEs) and represents the number of different potentially traumatic experiences a person has experienced before the age of 18. In previous literature, the ACE has been used in a range of ways, e.g., as a sum score, or truncated to an ordinal or dichotomous variable [1, 22, 23]. We planned to use the ACE sum score to test our hypotheses, assuming that higher numbers of ACEs are associated with poorer outcomes. Due to a highly skewed distribution of ACE scores in the final sample, we used a truncated ordinal version of the ACE variable with five levels (0, 1, 2, 3, ≥ 4 ACEs). The number of levels was chosen to ensure sufficiently large group sizes and was based on previous studies truncating the ACE variable [1].

Mental health status was operationalized by symptoms of major depressive disorder and symptoms of generalized anxiety disorder assessed by the screening tools Patient Health Questionnaire-9 (PHQ-9, German version [24]) and Generalized Anxiety Disorder-7 (GAD-7, German version [25]), respectively. The PHQ-9 comprises nine items assessing the key components of the DSM-IV criteria for a depressive episode on a four-point Likert scale (0 = not at all, 1 = several days, 2 = more than half of the days, 3 = nearly every day), referring to the occurrence of each symptom during the past two weeks. The GAD-7 comprises seven items assessing the key components of the DSM-IV criteria for a generalized anxiety disorder using the same instruction (past 2 weeks) and Likert scale as the PHQ-9. Both, PHQ-9 and GAD-7 have a reportedly high internal consistency (PHQ-9_α_ = 0.86–0.89; GAD-7_α_ = 0.89) [25, 26]. Intercorrelations of the GAD-7 with other mental health indices, as for example the PHQ-2 depression scale (*r* = 0.64) and Questionnaire on Life Satisfaction (*r* = −0.34), suggest construct validity [25]. Similarly, the PHQ-9 scores have been shown to correlate negatively with all SF-20 Health-related Quality of Life Scales. Especially the subscales previously being associated with depression, e.g. mental health and social subscale, have been demonstrated to decrease monotonically with increasing PHQ-9 scores [26]. Cases with missing data on the variables sex, age, SES-Index, or questionnaire data were excluded (n = 397).

#### Control variables

Research indicates that sex and SES are associated with differential ACE scores [1]. Data from a national survey in the United States of America (N = 211 376) showed that females report more ACEs than males (with a mean ACE score of 1.64 compared to 1.46, respectively) [27]. SES was investigated less frequently in the studies included in the meta-analysis, i.e., compared to 24 studies considering sex, only five studies included SES. However, the results indicate that individuals with higher SES are less likely to report ACEs (referent: high SES; OR_1ACE_ = 1.22, CI [1.12, 1.33]; OR_4+ACEs_ = 1.80, CI [2.62, 2.00]). Moreover, age [18], TIV, and sex [10, 11] are important control variables in any MRI study, since they have been shown to be associated with brain volume. Thus, sex, age, and SES as well as TIV were used as control variables in the analyses. In an earlier study we developed and validated a standardized SES-Index for the Hamburg City Health Study sample, which comprises three sub scores: education, job status, and income. The sub score education describes school and further education, while job status describes both the social prestige and responsibility of different types of work positions. The sub score income represents the current net equivalent income or, if applicable, the last income before retirement. Sub scores range between 1–7. The total SES-Index, which was used in this study, ranges from 3 to 21 [28].

#### MRI data acquisition and pre-processing

Images were acquired using a 3-T Siemens Skyra MRI scanner (Siemens, Erlangen, Germany), using a 32-channel head coil. For 3D T1-weighted anatomical images, rapid acquisition gradient-echo sequence (MPRAGE) was used with the following sequence parameters: repetition time (TR) = 2 500 ms, echo time (TE) = 2.12 ms, 256 axial slices, slice thickness (ST) = 0.94 mm, and in-plane resolution (IPR) = 0.83 × 0.83 mm^2^. Cases with missing data on MRI were excluded. For the ROI-analyses, cortical and subcortical segmentation was performed using the FreeSurfer7 toolbox [29, 30]. Cortical thickness or brain volume in regions of interest (rDLPFC, bilateral amygdala, and hippocampus) were extracted using the Destrieux Atlas [31] in FreeSurfer7 [29]. Right DLPFC thickness was determined as the mean of middle frontal gyrus and superior frontal gyrus [32]. For the exploratory VBM analyses, images were pre-processed and segmented into grey matter (GM), white matter, and cerebrospinal fluid using the Computational Anatomy Toolbox for the Analysis of Structural MRI Data (CAT12 [33]) in MATLAB (R2022b, [34]). We used default settings for cross-sectional data, whereby only GM was to be analysed, which was smoothed using a 6x6x6 kernel.

### Data analysis

#### ROI-based analyses

After quality checks, n = 228 cases (9%) were excluded [35]. Analyses were performed in JASP [36] and R [37] using Bayesian statistics which allow conclusions regarding evidence for the alternative and the null hypothesis and offer model selection tools (i.e., the Bayes Factor, BF) that quantify the evidence provided by the data. To first explore whether the expected associations that are logically necessary for a mediation were present in the given data, we performed two-tailed Bayesian correlation analyses with the variables of interest: ACE score, (aggregated) bilateral hippocampus and amygdala volumes, rDLPFC thickness, PHQ-9 score, GAD-7 score, age, sex, TIV, and SES-Index (default JASP prior P(M) = 1). Variables with expected significant correlational patterns were then tested for the hypothesized mediation effect. We planned to test the mediation hypothesis following “A Tutorial in Bayesian Potential Outcomes Mediation Analysis” [38]. Sensitivity analyses were performed using three modified datasets: (a) original sample excluding outliers, (b) original sample with imputed questionnaire data, (c) imputed sample excluding outliers. We applied multiple imputation by chained equations, with the criteria for imputation and outlier exclusion reported in Supplementary Table 2. We imputed behavioural data only when participants had provided at least one response on each questionnaire, and we refrained from imputing MRI data to minimize bias and preserve reproducibility. Results of the sensitivity analyses using (b) are reported here but results of (c) are only reported if they deviate from those of the original sample. Code of the analyses is publicly available (https://github.com/AnneKlimesch/hchs_ace.git).

#### Exploratory whole-brain analyses (Voxel based Morphometry)

In addition to the results of the ROI-analyses, we explored differences in regional grey matter volume (rGMV) at a whole brain level comparing five subgroups that were based on the truncated categorical ACE variable: 0 ACE, 1 ACE, 2 ACEs, 3 ACEs, 4 or more ACEs (referred to as 4+ in the following). A voxel-based morphometry (VBM) [39] analysis was conducted using the statistical parametric mapping software 12 (SPM12, v7771 [40]). Toolboxes were run in MATLAB (R2022a, [34]). We entered sex, age, and TIV as covariates. We performed multiple two-sample t-tests comparing the groups with at least one ACE (ACE_1_, ACE_2_, ACE_3_, and ACE_4+_) to the ACE_0_ group in CAT12 (CAT12.7, version 2734) using the default settings, absolute threshold masking of 0.1 (implicit masking = yes), and an uncorrected threshold of *p* < 0.001 for the resulting maps in SPM12 (version 7771). To correct for multiple comparisons, spmT maps were FWE-thresholded at peak-level in addition to using the statistical extent threshold (expected voxels per cluster), which in CAT12 is combined with a non-isotropic smoothness correction based on permutation as proposed by Hayasaka and Nichols [41]. Contrasts were tested in both directions, i.e., the ACE_1_, ACE_2_, ACE_3_, and ACE_4+_ groups were contrasted both to being larger than ACE_0_ and smaller than ACE_0_ concerning differences in rGMV. After identifying significant clusters using the above-described approach, GM probability (mean values, no scaling) was extracted from each of the previously identified significant clusters, using the REX tool version 2.1 in MATLAB.

## Results

The sample of analysis comprised n = 1 900 participants (42% female) with a comparatively high SES-Index (see Supplementary Table 3), as evidenced by comparison with a nationally representative German health study [42], likely reflecting the high prosperity of the city of Hamburg among German cities [28]. The mean net equivalent income of the sample (2166.67€ – 2374.99€) was similar to that of the general population in Hamburg from 2016–2022 (2113,38€) [43]. The sample had a highly skewed distribution of ACE scores (Supplementary Table 4). The descriptive statistics for the final sample n = 1 900 are displayed in Table 1. Frequencies for variables of interest are reported in Supplementary Tables 1 and 4. Males reported generally lower levels of ACEs (M = 0.83, SD = 1.17) than females (M = 1.21, SD = 1.40). Similarly, males had lower mean PHQ-9 scores (M = 2.92, SD = 3.29) and GAD-7 scores (M = 2.19, SD = 2.69) than females (M_PHQ-9_ = 4.06, SD_PHQ-9_ = 3.79; M_GAD-7_ = 3.09, SD_GAD-7_ = 3.24). We compared the ACE frequencies with the ageing sub-sample of the German NAKO public health cohort study [44] and report the results below Supplementary Table 4. In addition, we compared the sample characteristics (age, sex, SES-Index, ACE-score, PHQ-9 score, GAD-7 score) to those participants of the cohort study who chose not to participate in the MRI scan (see Supplementary Table 5). Differences with small to moderate effect sizes were found for age and sex, with people participating in the MRI scan being older and more often male than female. Participants who underwent MRI also had a slightly higher SES-Index and slightly lower ACE-, PHQ-9, and GAD-7 scores, though related effect sizes were below 0.1. Supplementary Table 6 displays the descriptives statistics of the sample with imputed questionnaire data (n = 2 042) which was used for sensitivity analyses.

**Table 1:** Descriptives and frequency statistics of the study sample (1 900).

		Total sample %	Male % (n = 1 097)	Female % (n = 803)
**Age group**^**1**^ **(years)**	**mean (sd)**	63.46 (8.38)	63.66 (8.26)	63.19 (8.54)
46–55		21.3	19.8	23.3
56–65		31.8	32.9	30.2
66–75		42.2	42.3	42.2
≥78		4.7	4.9	4.4
**SES-Index**	**mean (sd)**	12.95 (3.50)	13.52 (3.54)	12.15 (3.28)
3–8		6.1	5.5	7.3
9–15		65.1	59.2	73.2
16–21		28.6	35.4	19.4
**ACE score (dichot.)**				
0		50.4	54.5	44.8
1–10		49.6	45.5	55.2
**PHQ-9 score**	**mean (sd)**	3.40 (3.55)	2.92 (3.29)	3.79 (3.09)
0–4		71.6	76.6	64.9
5–9		21.1	18.1	25.2
10–14		5.7	4.3	7.7
15–19		1.4	1.1	1.7
20–27		0.2	0.0	0.5
**GAD-7 score**	**mean (sd)**	2.56 (2.97)	2.18 (2.69)	3.09 (3.24)
0–4		80.0	84.8	73.5
5–9		16.9	13.1	22.0
10–14		2.2	1.5	3.1
15–21		0.9	0.6	1.4

^1^Participants were born 1938 and later.

### ROI-based analyses

The Bayesian correlation analysis (Table 2) revealed strong evidence in favour of a positive association between the ACE and PHQ-9 score (r = 0.324, BF_10_ = 1.871 × 10^49^) as well as the GAD-7 score (r = 0.217, BF_10_ = 1.383 × 10^42^). However, correlations between ACE score and ROIs (rDLPFC thickness, amygdala, and hippocampal volume), as well as brain variables and the outcomes (PHQ-9, GAD-7) yielded near-zero correlation coefficients with moderate to very strong evidence in favour of the null hypothesis or no evidence of any correlation. These relationships were additionally tested for non-monotonic relationships using scatterplots and generalized additive models. The latter indicated a weak non-monotonic relationship between bilateral amygdala volume and GAD-7 (Supplementary Table 7) while the scatterplots did not. Since the smooth term of the generalized additive models was close to 1, indicating a linear approximation of the model, a linear relationship was assumed despite the significant generalized additive model.

**Table 2:** Bayesian intercorrelations of variables of interest.

		ACE score	PHQ-9 score	GAD-7 score	hippocampus volume	amygdala volume	rDLPFC thickness	age	SES-Index	sex	TIV
ACE score	T	-									
	^BF^10	-									
PHQ-9 score	T	0.234***	-								
	^BF^10	1.871x10^49^	-								
GAD-7 score	T	0.217***	0.595	-							
	^BF^10	1.383x10^42^	∞	-							
hippocampus volume	T	−0.006	−0.027	0.003	-						
	^BF^10	0.033	0.143	0.031	-						
amygdala volume	T	−0.041	−0.039	−0.035	0.539***	-					
	^BF^10	1.109	0.742	0.435	1.925x10^267^	-					
rDLPFC thickness	T	0.030	0.021	0.039	0.064**	0.072***	-				
	^BF^10	0.219	0.074	0.739	202.830	1921.784	-				
age	T	−0.065***	−0.086***	−0.107***	−0.297***	−0.267***	−0.169***	-			
	^BF^10	279.740^a^	183886.768	1.065x10^9^	1.598x10^80^	3.057x10^64^	6.682x10^24^	-			
SES-Index	T	−0.083***	−0.072***	−0.040	0.141***	0.148***	0.001	−0.080***	-		
	^BF^10	76336.210	1827.158	0.855^a^	6.912x10^16^	5.434x10^18^	0.030	29601.512	-		
sex	T	0.120***	0.153***	0.136***	−0.262***	−0.346***	0.103***	−0.020	−0.160***	-	
	^BF^10	5.356x10^11^	1.388x10^20^	4.997x10^15^	9.414x10^61^	1.776x10^109^	1l.797x108	0.068	1.718x10^22^	-	
TIV	T	−0.056*	−0.064***	−0.066***	0.380***	0.413***	−0.068***	−0.040	0.176***	−0.525***	-
	^BF^10	26.944^a,b^	187.759	305.056	9.341x10^131^	3.017x10^150^	513.995	0.962	1.028x10^27^	3.420x10^253^	-

*BF_10_ > 10, ** BF_10_ > 30, *** BF_10_ > 100.

T Kendall’s tau.

^a^These relationships did not have a BF_10_ > 10 in the sensitivity analysis using the complete cases sample excluding outliers (n = 1 815).

^b^These relationships did not have a BF_10_ > 10 in the sensitivity analysis using the imputed sample excluding outliers (n = 1 945).

**Fig. 2:**
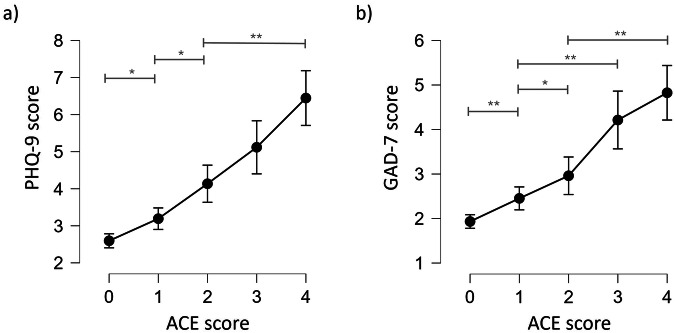
Relationship between the ordinal ACE variable and PHQ-9 as well as GAD-7 scores, controlling for covariates. For both panels **a**) and **b**), ACE score level 4 comprises individuals reporting 4–10 adverse childhood experiences. *BF > 20. **BF > 30.

Since the results of these analyses did not endorse the performance of the hypothesized mediation models, we focused on the exploration of the significant relationship between ACE score and mental health. We performed two Bayesian ANCOVAs on the associations between ACE and PHQ-9 and GAD-7 to disentangle the relationship between the number of ACEs and the severity of depression and anxiety under consideration of age, sex, and SES as covariates. We used uniform model priors and weakly informative coefficient priors - the latter to avoid overfitting. To confirm this choice, we performed prior sensitivity analyses comparing four combinations of model and coefficient priors for each of the Bayesian ANCOVAs testing the main pre-registered hypothesis that ACEs are positively associated with PHQ-9 scores and GAD-7 scores. In the prior sensitivity analyses, the key findings remained stable across analyses (Supplementary Table 8). Results concerning the PHQ-9 suggested strong evidence for the H1 (BF_10_ = 3.769 × 10^36^), indicating that depressive symptoms (PHQ-9 score) increase per level of ACEs. The mean PHQ-9 sum score was 2.60 (SD = 2.99) for individuals with ACE_0_ compared to 6.45 (SD = 4.74) for individuals with ACE_4+_ (Fig. 2a). Post-hoc comparisons between all ACE score levels yielded strong or very strong evidence in favour of H1 for all comparisons, except those between ACE_2_ and ACE_3_ as well as ACE_3_ and ACE_4+_ (Supplementary Table 9).

The ANCOVA of GAD-7 scores yielded strong evidence in favour of the H1 (BF_10_ = 3.129 × 10^30^), suggesting that anxiety symptoms (GAD-7 score) increase per level of ACEs. The mean GAD-7 sum score was 1.93 (SD = 2.41) for individuals with ACE_0_ compared to 4.82 (SD = 3.90) with ACE_4+_ (Fig. 2b). Post-hoc comparisons between all ACE score levels yielded strong or very strong evidence in favour of H1 for all comparisons, except those between ACE_1_ and ACE_2_ as well as ACE_3_ and ACE_4+_ (Supplementary Table 10). Supplementary Tables 11–16 display the results of the three sensitivity analyses using the samples: a) complete cases excluding outliers (n = 1 815), b) imputed data (n = 2 042), c) imputed data excluding outliers (n = 1 945). These results are in line with the main analysis suggesting that depressive symptoms (PHQ-9 score) as well as anxiety symptoms (GAD-7) increase per level of ACEs.

### Exploratory whole-brain analyses (Voxel-based Morphometry)

#### Differences in rGMV between groups ACE_1_ and ACE_0_

There were n = 958 participants in the ACE_0_ sample which was compared to the n = 448 ACE_1_ sample (i.e., individuals who had experienced a single adverse childhood experience) concerning possible differences in rGMV. No significant clusters indicating rGMV differences between the groups were identified (FWE-corrected peak-level p-values, cluster extent threshold = expected voxels per cluster). The ACE_0_ sample constitutes the same no ACEs reference group for all following comparative analyses.

#### Differences in rGMV between groups ACE_2_ and ACE_0_

The ACE_2_ sample (n = 207) was compared to the ACE_0_ sample: no significant clusters indicating rGMV differences between the groups were identified (FWE-corrected peak-level p-values, cluster extent threshold = expected voxels per cluster).

#### Differences in rGMV between groups ACE_3_ and ACE_0_

**Fig. 3:**
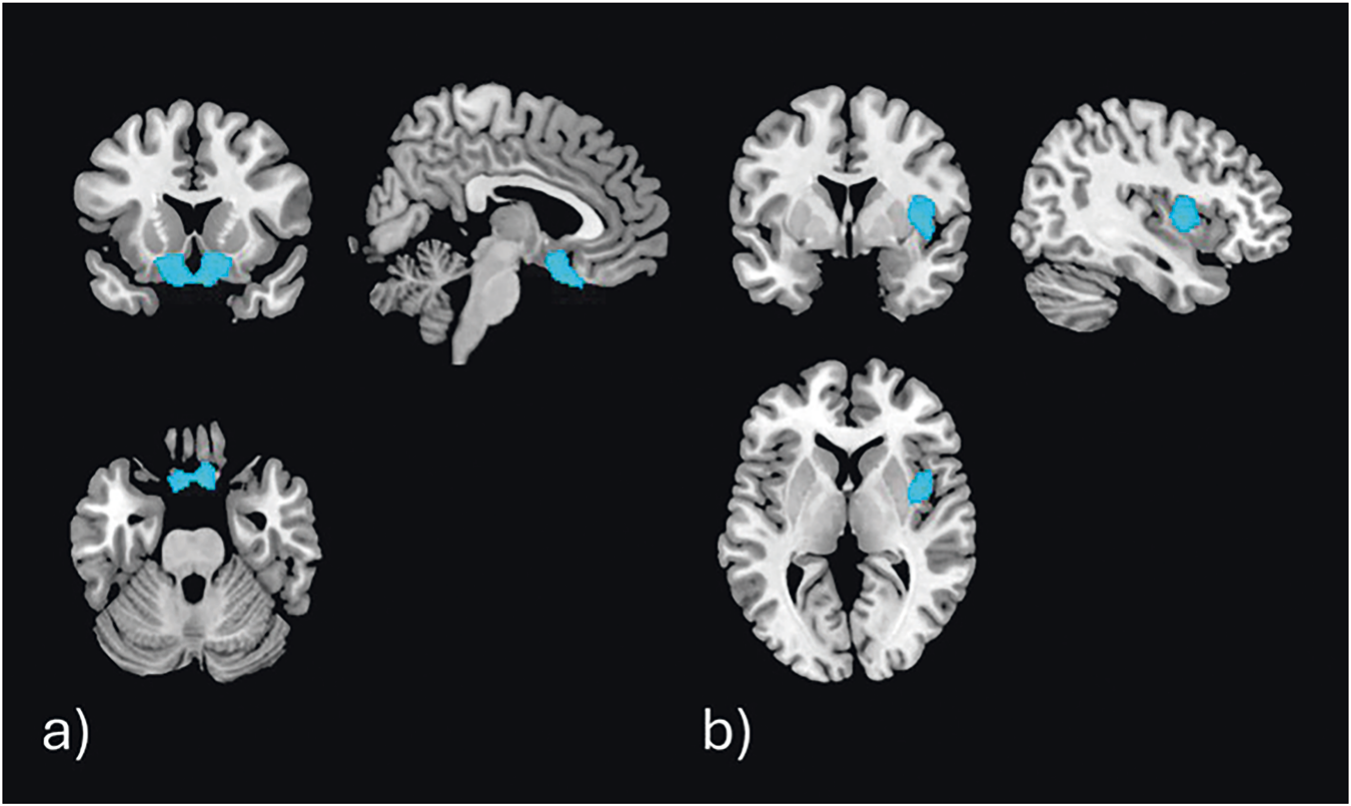
Depiction of the two largest significant clusters identified to exhibit lower rGMV in individuals with ACE_3_ compared to ACE_0_. **a** shows a cluster (*k* = 2 365, *p* = 1.1e-06, cluster peak MNI coordinate: 4, 16, −26) that mostly comprises the ventral striatum with bilateral NAcc and olfactory bulb. **b** shows a cluster (*k* = 815, *p* = 1.5e-06, cluster peak MNI coordinate: 39, 2, 4) mainly comprising the right insula.

The ACE_3_ sample (n = 126) was compared to the ACE_0_ sample: no significant differences concerning the contrast rGMV ACE_3_ > ACE_0_ were identified, suggesting that individuals with 3 ACEs had no regions of higher rGMV compared to individuals without ACEs. However, six clusters of reduced rGMV in ACE_3_ compared to ACE_0_ emerged (FWE-corrected peak-level p-values, cluster extent threshold *k* > 38). The largest cluster (k = 2 365, p = 1.1e-06, cluster peak MNI coordinate: 4, 16, −26) comprised parts of the left and right gyrus rectus, nucleus accumbens (NAcc) and olfactory bulb (see Fig. 3a). The second largest cluster (k = 815, p = 1.5e-06, cluster peak MNI coordinate: 39, 2, 4) mainly comprised the right insula (Fig. 3b). A smaller cluster (k = 180, p = 7.6e-06, cluster peak MNI coordinate −39, 36, −9) comprised the left inferior frontal gyrus and posterior orbital gyrus. A cluster including the right amygdala (k = 98, p = 1.1e-05, cluster peak MNI coordinate: 21, −4, −10) was also identified. Lastly, two small clusters, one in the right superior frontal gyrus – dorsolateral (k = 48, p = 1.4e-05, cluster peak MNI coordinate: 10, 52, 28) and another in right middle frontal gyrus (k = 41, p = 1e-05, cluster peak MNI coordinate: 36, 52, −9) were identified.

#### Differences in rGMV between groups ACE_4+_ vs. ACE_0_

The ACE_4+_ sample (n = 161) was compared to the ACE_0_ sample and again, no significant differences concerning the rGMV contrast ACE_4+_ > ACE_0_ were identified, suggesting that individuals with 4 or more

**Fig. 4:**
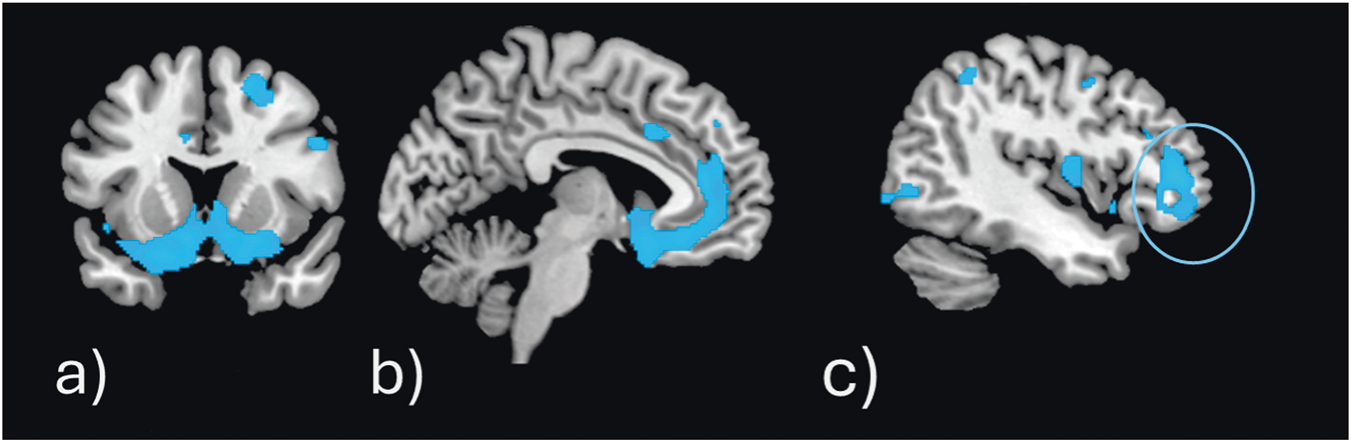
Depiction of the two largest significant clusters identified to exhibit lower rGMV in individuals with ACE_4+_ compared to ACE_0_. **a** shows a widespread cluster (*k* = 11 036, *p* = 7.7e-08, cluster peak MNI coordinate: 26, 40, 42) that mostly comprises the ventral striatum with bilateral NAcc and olfactory bulb, and (**b**) the anterior and subgenual cingulate cortex. **c** shows the second largest identified significant cluster (*k* = 1 658, *p* = 6.7e-07, cluster peak MNI coordinate: −44, 42, 6) mostly comprising the triangular part of inferior frontal gyrus.

ACEs had no regions of enhanced rGMV compared to controls with no ACEs. However, for the opposite contrast (ACE_4+_ < ACE_0_), a total of 22 significant (FWE-corrected peak-level p-values, expected voxels per cluster k > 37) clusters were identified. Supplementary Table 17 shows all clusters with p-values, cluster sizes, cluster peak MNI coordinates, and assigned regions ordered by descending cluster size. The two largest clusters are shown in Fig. 4. These comprised a large cluster involving different areas of the brain, namely right superior frontal gyrus – dorsolateral, extending to the anterior cingulate cortex, bilateral NAcc and olfactory bulb (k = 11 036, p = 7.7e-08, cluster peak MNI coordinate: 26, 40, 42; see Fig. 4a and b which capture the cluster at the ventral striatum and pre-/ subgenual anterior cingulate cortex). The second largest cluster (k = 1 658, p = 6.7e-07, cluster peak MNI coordinate: −44, 42, 6) was mainly located in the left inferior frontal gyrus – triangular part (see Fig. 4c).

The remaining significant clusters of reduced rGMV in individuals with ACE_4+_ compared to ACE_0_ are summarized in Supplementary Table 17. Strikingly, there were bilateral patterns of reduced rGMV: Frontal lobe rGMV reductions were present bilaterally in the frontal gyrus, (medial) orbital gyrus, and middle frontal gyrus as well as in the left precentral gyrus and Rolandic operculum. There were bilateral rGMV reductions in the temporal lobe in both middle and superior temporal gyrus, as well as in the right fusiform gyrus. Parietal lobe lower volumes were left lateralized with the inferior parietal gyrus showing reduced rGMV. Occipital lobe rGMV reductions were visible bilaterally in inferior occipital gyrus. Bilateral reductions in rGMV were apparent in anterior cingulate cortex, NAcc, olfactory bulb and gyrus rectus. Insular volumetric reductions were left lateralized. Finally, cerebellar rGMV reductions were bilaterally observed in lobule VIII (left) and VI (right).

Based on these results we extracted the mean peak values of the described GMV clusters using region of interest extraction (REX) and aimed to test for a mediation effect of these on the relationship between ACE and mental health. However, after controlling for TIV, age, sex, and SES-Index, there was no evidence for a relationship between these clusters and PHQ-9 as well as GAD-7.

## Discussion

The present study tested if ACE associated deviations in regional cerebral structure might persist into old age and are thus identifiable in a large (n = 1 900) sample of randomly sampled older adults (46–78 years) from Hamburg, Germany. In addition, we hypothesised a mediation of these brain structural deviations on the relationship between ACEs and mental health in mid to old age. Structural deviations of predefined ROIs (amygdala, hippocampus, rDLPFC) were not confirmed, however, the relationship between ACE and mental health (depressive symptoms, anxiety symptoms) which is well-established in young and middle age was replicated in the ageing sample. Moreover, a whole-brain analysis revealed widespread reductions but no increases in rGMV in individuals with three, four, or more ACEs in regions associated with executive functioning, cognitive control, reward processing, processing of emotions, and emotion regulation. The study has substantially more power than most previous MRI studies which demonstrates its value to the field of ACE research. Moreover, the sample represents the general population of Hamburg as well as a comparably old age group, which has rarely been studied in the context of ACE so far [10].

The finding that structural deviations in ROIs in association with ACE were not replicated in this ageing sample offers multiple interpretations. On the one hand, based on the lack of previous research in ageing populations, one could assume that structural deviations in ROIs outgrow over the years - an argument which is, however, weakened by the consistently strong association between ACEs and mental health outcomes in the present study. On the other hand, the choice of ROIs may have been biased by the assumption that the operationalization of ACE is coherent across literature. Also, it has been demonstrated that there is considerable heterogeneity in childhood adversity questionnaires across studies [17, 35]. Only a subset of previous studies has employed the ACE-questionnaire or a validated scale assessing the same underlying construct. Despite these considerations, we performed an initial ROI-analysis, since a targeted hypothesis was clearly deductible from previous literature and superior to a mere exploratory analysis [10]. In sum, most likely the results of the ROI-analysis can be explained by a lack of comparability with studies the ROIs were based on and more specifically with the operationalization of ACE in the literature.

In comparison to the ROI-analysis, the strong association between ACE and depressive symptoms (PHQ-9) as well as anxiety symptoms (GAD-7) was in line with findings of previous studies [1, 6]. The results show that in the ageing general population of Hamburg the number of different ACEs associates with depressive symptoms and anxiety symptoms: the PHQ-9 sum score increased 0.6–3.0 points per level of ACE while the GAD-7 sum score increased 0.5–1.3 points per level of ACE. Thus, the cumulative effect of ACEs on mental health persists into mid and late adulthood. The increases in mean depression and anxiety symptom scores may seem small, considering that the PHQ-9 sum score ranges from 0–27 [24] and the GAD-7 from 0–21 [25], however, the study sample was derived from the general population of Hamburg instead of a clinical sample and thus generally displays low mean scores on mental disorder screenings. This study presents strong evidence that the negative association between ACEs and mental health pertains to late adulthood which renews the question which (neural) mechanisms are at work that carry these effects into old age despite the diversity of additional influencing factors across the lifespan.

The VBM revealed distributed frontal and limbic GMV reductions in subgroups with 3 versus 0 ACEs, and 4–10 versus 0 ACEs, however differences were not observed for those with 1–2 ACEs. This suggests that there may be an adversity severity threshold for the impact of ACEs upon brain structure – at least into middle-to-old age. In addition, six regions of reduced GMV were found to overlap considerably for the ACE_3_ as well as ACE_4+_ group: bilateral gyrus rectus, bilateral olfactory bulb, right middle frontal gyrus, bilateral NAcc, right insula, and left inferior frontal gyrus. The latter two align with findings of a voxel-wise meta-analysis involving young to mid-old samples with ACE [18] while reductions in the NAcc settle with VBM results in adolescents [11]. Similarly, the study using the UK Biobank (N = 6 751) reported reduced ventral striatum volumes in adults with experiences of emotional abuse in childhood [16]. These results hint at a long-term effect of ACEs on GMV in the ventral striatum, the reward system of the brain [45], the insula, involved in affective processing, interoception, and social cognition [46], as well as the left inferior frontal gyrus, which associates with attention regulation [47]. If reductions in NAcc volume evolve shortly after exposure to ACEs, they may represent the initial brain structural response to ACEs, which has hypothetically behavioural as well as neural consequences in adulthood. If instead reductions in the NAcc exist before exposure to ACEs, they may represent a vulnerability to the development of mental disorders following ACEs.

Individuals with 4–10 ACEs exhibited additionally more widespread GMV reductions in frontal and limbic regions. The wide distribution of clusters may be explained by a diversity of factors that were influenced by the ACEs and/or acted on GMV over the life course. The finding that these widespread reductions were found in the group with four or more ACEs parallels behavioural studies claiming that this marks the number of ACEs which associates particularly strongly with a diversity of negative (mental) health outcomes [48–50]. The limbic system being part of these widespread reductions is, e.g., implicated in emotion regulation, memory processing, and stress responses [51] has been associated with mental disorders, such as depression and anxiety [11, 43]. This aligns with the association between ACEs and depression and anxiety symptoms in the present sample. The study suggests that the effect of ACEs on brain structure could last until or evolve into old age, with the scale of effects depending on the number of ACEs.

The question remains if some of the identified lower rGMV represent vulnerabilities that enhance the effect of ACEs or whether they are consequences of ACEs, or both. In this context, the concept of brain reserve may offer an alternative explanatory framework. Unlike region-specific structural alterations, brain reserve refers to the broader neurobiological capacity—across multiple levels of brain organization—that supports resilience against stressors, pathology, and ageing [52, 53]. It includes both innate and developmentally acquired structural resources that help maintain cognitive and emotional functioning despite adverse exposures. Individuals with higher brain reserve may be more resilient to the structural and functional consequences of ACEs, potentially delaying or mitigating the onset of psychopathology until a critical threshold is reached. Conversely, ACEs experienced early in life may compromise the development of brain reserve, thereby increasing vulnerability over time. Future longitudinal studies are needed to clarify whether reduced GMV reflects diminished brain reserve established early in life, a lack of neuroprotective factors, neurodegeneration or a cumulative consequence of several mechanisms.

A different interpretation of the presented results may be that the associations between ACEs and mental health in late adulthood as well as between ACEs and brain related changes in late adulthood are completely independent processes. However, based on previous literature consistently reporting associations between ACEs and brain-related aberrances in adulthood [7, 10, 11], it can be assumed that neural mechanisms are more complex than the ones investigated in the present study. The rejection of the presented statistical model is no proof of independence of the tested associations. While addressing the limitations of the present dataset, future studies should still assess the relationship between childhood trauma, brain alterations, and mental health in (late) adulthood. It should be considered that these pathways may oppose those in our original hypothesis, e.g., mental health symptoms influencing both the perception or reporting of past ACEs and brain structure.

The limitations to be addressed in the future are the following: First, we analysed cross-sectional data to infer a process that would ideally require a longitudinal design. Consequently, ACEs were operationalized via self-report measures at the time of data collection. While this approach is commonly used in ACE research for reasons of feasibility [54], it introduces the potential for recall bias. Dube et al. found that ACE self-reports are reasonably stable in adulthood [55], which challenges the concern that ACE reports are biased by the age of our participants. However, relative stability of reporting ACE across adulthood does not necessarily imply these reports truly reflect what was experienced in childhood. Hence, the limitation of report bias should be considered, and it might be tackled by large-scale longitudinal health data in the future, or by validating the self-report ACE scale collected in adulthood against third-person reports or medical/ social service records. Unfortunately, such data were not collected in the present study. This leads to a related limitation concerning the measurement of mental health outcomes using cross-sectional data. As with ACEs, the study design restricts the extent to which we can account for dynamic, time-dependent influences on mental health across the lifespan. One class of such influences is medical comorbidities, which can fluctuate and impact mental health in complex ways. The number of medical comorbidities that could act as confounders can be large and controlling for these in the given cross-sectional setting would be vague, justifying separate studies to investigate their relationship with mental health outcomes in detail.

Second, the ACE-questionnaire is, due to its shortness, less informative than, e.g., the Childhood Trauma Questionnaire (which includes the frequency and severity of ACE); however, it is more efficient considering the scale of a cohort study. Similarly, the PHQ-9 and GAD-7 may lack the sensitivity to fully capture the spectrum of depressive and anxiety symptoms. In general populations, both scales show floor effects and thus have limited sensitivity for mild or subclinical cases. However, considering their high internal consistency and reported construct validity, they represent an inevitable trade-off between efficiency and validity in the context of a cohort study.

Third, we made the ad-hoc decision to analyse the ACE score as an ordinal variable, establishing five levels based on the distribution of data and ambiguous information in previous literature. In general, the operationalization and scaling of ACEs can be considered a problem in ACE literature, due to its variability, and our approach reflects the absence of an established standard. The 28-item long Childhood Trauma Questionnaire as well as the ACE are frequently used in international literature with the latter often being adapted, e.g., comprising a varying number between 8-11 items, and differing response scales [39–42] while other studies use non-validated questionnaires [10]. These deviations lead to heterogeneity in the computation and interpretation of the ACE score, which eventually interferes with the validity of conclusions in ACE research. This issue has scientifically been addressed [54, 56] and should be considered in the development of future studies on ACE to establish common standards, assuring comparability across studies.

Fourth, we assumed the data being missing not completely at random (MNCAR), which is relevant in mental health research where non-random missingness is common [57]. Individuals with depressive or anxiety symptoms may be less likely to participate or to complete behavioural assessments, introducing a potential selection bias. In our study, approximately 15% (n = 397) of the initial sample were excluded due to missing data on questionnaires. This attrition may disproportionately affect individuals with higher mental health burden, potentially leading to an underestimation of associations between ACEs and mental health outcomes [57]. Although we used non-imputed data for our primary analyses to avoid inflating potentially biased patterns, we conducted sensitivity analyses using multiple imputation to assess the robustness of our findings. These complementary approaches confirmed our main findings and help account for, but cannot eliminate, the risk of bias introduced by data not missing at random. This is underscored by the restricted scope of our imputation. We refrained from imputing MRI ROI data to avoid bias due to the complex nature of structural measures and excluded cases with fully missing SES or primary behavioural variables to prevent imputation of data using insufficient information. Consequently, the number of imputations was limited and the sample size of the imputed dataset only slightly exceeded that of the complete-case dataset.

Fifth, the analysis included only participants who opted in for the MRI scan. Reasons for non-participation can be manifold and potentially biasing but were not explored. Our comparison of available variables showed that MRI participants were older and more often male. Differences in SES-Index, ACE, and symptoms of depression and anxiety were minimal, though it remains plausible that MRI participants were generally healthier and thus better able to (also physically) tolerate the scan. Given the small differences observed, we do not assume substantial bias but in future research we would aim to match participants on key variables, reduce reliance on opt-in scans by encouraging full participation, or at least document reasons for opting out.

Apart from methodological considerations, the present results encourage a close-up analysis of the associations of different ACEs in future studies. Subgroups matched by type of ACE (i.e., physical or verbal abuse, physical or emotional neglect, household dysfunction) should be compared concerning mental health outcomes and brain structure across lifetime. Based on the assumption that more severe forms of ACEs, such as abuse and neglect, correlate with other forms of household dysfunction, one may expect that outcome measures of individuals exposed to abuse and/or neglect resemble those of the ACE_4+_ group in the present analysis. However, a type- or class-based approach to ACEs could uncover distinct effects of different ACEs on mental health and brain structure. In addition, analysis of cohort data from the general population could focus on external and internal protective factors, such as SES and brain reserve. Future research should explore the role of brain reserve, for example as a mediating factor between ACEs and mental health outcomes, or as a moderator that shapes individual vulnerability to the effects of ACEs. In addition, our initial mediation hypothesis would be worthwhile to test in longitudinal data [6], including the key clusters identified in the present study as mediators, because the present results may hint at lifelong and potentially dynamic effects of ACEs on mental health and brain structure. These approaches could inform the development of interventions such as targeted cognitive training, early ACE detection and social support strategies, or psycho-therapeutic approaches [58–60]. Eventually, genetic data could be considered to discover vulnerability and resilience factors of ACEs on a biological level and associate these with behavioural as well as neural outcomes across the lifespan.

Finally, adverse childhood experiences are common in the general population, and their numbers seem to have increased during the Covid-19 pandemic. Similarly, large-scale events, such as, the climate crisis and wars, will cause traumatic events for children, adolescents, and adults around the world. There is an inherent need to understand the long-term effects of ACEs as well as the risk factors for these long-term effects concerning mental and brain health, to develop focused prevention and intervention strategies. This study demonstrates that especially exposure to four or more ACEs negatively associates with mental health as well as GMV in frontal and limbic brain areas across one’s adult life into old age. Since the world-population is ageing and mental health in old age is a critical topic for societies already, we need to further understand how ACEs contribute to psychiatric risks.

## Supplementary information

Supplementary Information

## Data Availability

The data that support the findings of this study are available from the Hamburg City Health Study Center, but restrictions apply to the availability of these data, which were used under license for the current study, and so are not publicly available. Data are however available from the co-author, L. Ascone (l.ascone-michelis@uke.de), upon reasonable request and with permission of the Hamburg City Health Study Center. Code of the analyses is publicly available (https://github.com/AnneKlimesch/hchs_ace.git).
