# Supplementary material for: Echoes of childhood trauma: the relationship between adverse childhood experiences, brain structure, and mental health in aging adults
Source: Transl Psychiatry. 2026 Feb 3;16:52. doi: 10.1038/s41398-026-03811-2 (PMC12873319; doi:10.1038/s41398-026-03811-2)
Supplement: Supplementary file 1 — Supplementary Information [file 41398_2026_3811_MOESM1_ESM.docx]

**Supplementary Information**Manuscript: Echoes of childhood trauma: The relationship between adverse childhood experiences, brain structure, and mental health in aging adults

**
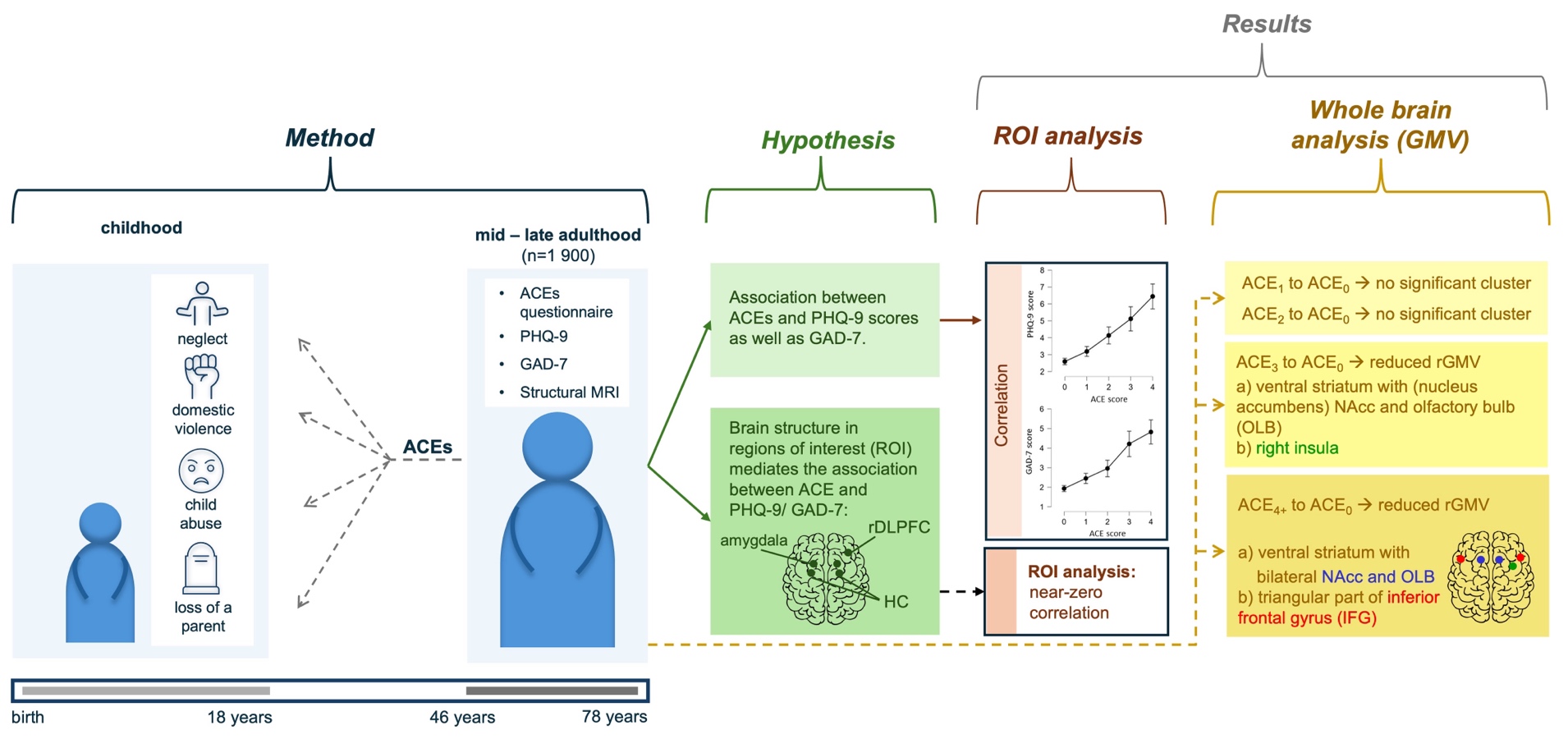
**

**Figure 1.** Graphical abstract.

| **Supplementary Table 1.** Overview of the ACE items and frequencies in the sample  (n = 1 900). | |
| --- | --- |
| **Items (concerning experiences before the 18^th^ birthday)** | **n (%)** |
| 1. Did a parent or other adult in the household…   …often or very often swear at, insult, or put you down? OR  …often or very often act in a way that made you afraid that you would be physically hurt? | 292 (15.4) |
| 1. Did a parent or other adult in the household…   …often or very often push, grab, shove, or slap you? OR  …often or very often hit you so hard that you had marks or were injured? | 176 (9.3) |
| 1. Did an adult or person at least 5 years older ever…   …touch or fondle you in a sexual way? OR  …have you touch their body in a sexual way? OR  …attempt oral, anal, or vaginal intercourse with you? OR  …actually have oral, anal, or vaginal intercourse with you? | 145 (7.6) |
| 1. Did you feel often or very often that…   ...no one in your family loved you or thought you were special? OR  …your family members did not look out for each other, did not feel close to each other, or did not support each other? | 281 (14.8) |
| 1. Did you feel that…   …you didn’t have enough to eat, had to wear dirty clothes, or had no one to protect or take care of you? OR  … your parents were too drunk or "high" to look after you or take you to the doctor when you needed it? | 56 (2.9) |
| 1. Have you ever lost a biological parent through divorce, by them leaving you, or for other reasons? | 415 (21.8) |
| 1. Was your mother (or stepmother) …   …sometimes, often, or very often pushed, grabbed, slapped, or had something thrown at her? OR  …sometimes, often, or very often kicked, bitten, hit with a fist, or hit with something hard? OR  …ever repeatedly hit over at least a few minutes? OR  …ever threatened with, or hurt by, a knife or gun? | 91 (4.8) |
| 1. Did you   …live with anyone who was a problem drinker or alcoholic? OR …live with anyone who used street drugs? | 303 (15.9) |
| 1. Was a household member depressed or mentally ill? OR Did a household member attempt suicide? | 263 (14.1) |
| 1. Did a household member go to prison? | 40 (2.1) |
| *Note.* These frequencies are generally comparable to another representative German sample (n = 2 531) (2), whereby questions 1, 2, 3, 4, 6, and 9 are 0.2 - 3.5 percent points higher in the present sample and questions 5, 7, 8, and 10 are 0.8 – 5.0 percent points lower in the present sample. This table displays the English version of the questionnaire [Reference: Felitti, V. J., Anda, R. F., Nordenberg, D., Williamson, D. F., Spitz, A. M., Edwards, V., Koss, M. P., & Marks, J. S. (1998). Relationship of childhood abuse and household dysfunction to many of the leading causes of death in adults. The Adverse Childhood Experiences (ACE) Study. American Journal of Preventive Medicine, 14(4), 245–258. https://doi.org/10.1016/s0749-3797(98)00017-8]. | |

| **Supplementary Table 2.** Criteria for imputation and outlier exclusion | | | |
| --- | --- | --- | --- |
| **Details of Imputation procedure** | | | **n** |
|  | 1. Cases were excluded when: ^1^ | |  |
|  |  | Missing data on SES-Index variable ^2^ | 102 |
|  |  | 100% missing data on all questionnaire items (PHQ-9, GAD-7, ACE) | 119 |
|  |  | 100% missing data on all PHQ-9 items | 10 |
|  |  | 100% missing data on all GAD-7 items | 10 |
|  |  | 100% missing data on all ACE items | 14 |
|  | 1. Imputer variables: age, sex, existing questionnaire items, SES-Index sub scores, individual income | |  |
|  | 1. Imputed variables: missing questionnaire values, if at least one item answered | |  |
|  | 1. Imputation method: predictive mean matching and logistic regression ^3^ | |  |
| **Criteria for outlier exclusion** | | | |
|  | Values of the questionnaire sum scores that were 3 times the standard deviation above or below the mean. | | 85^4^  91 |
| *Note. ^1^* Missing data were removed in the presented order. The numbers are thus additive. ^2^ SES-data were not imputed, because they have been imputed in a previous study which used the whole behavioural dataset of n = 10 000 cases for the imputation (Klimesch et al. 2024). ^3^ Depending on the structure of the variable; ^4^ The first value pertains to the sample pre-imputation (main sample of analysis); the second value pertains to the sample after imputation. | | | |

| **Supplementary Table 3.** SES-Index in the Hamburg City Health Study sample (n = 1 900). | | | | |
| --- | --- | --- | --- | --- |
| **Category** | **Quintile of SES** | **Lower boundary** | **Upper boundary** | **n (%)** |
| **“low”** | *1^st^ quintile* | 3.3 | 9.8 | 380 (20.00) |
| **“medium”** | *2^nd^ quintile* | 9.9 | 11.5 | 383 (20.16) |
|  | *3^rd^ quintile* | 11.6 | 13.3 | 377 (19.84) |
|  | *4^th^ quintile* | 13.4 | 16.2 | 386 (20.32) |
| **“high”** | *5^th^ quintile* | 16.3 | 21.0 | 374 (19.68) |

| **Supplementary Table 4.** Distribution of ACE scores in the sample (n = 1 900). | | | | | | | | | | | |
| --- | --- | --- | --- | --- | --- | --- | --- | --- | --- | --- | --- |
| **ACE score** | **0** | **1** | **2** | **3** | **4** | **5** | **6** | **7** | **8** | **9** | **10** |
| **n** | 958 | 448 | 207 | 126 | 68 | 48 | 23 | 7 | 9 | 4 | 2 |
| **%** | 50.42 | 23.58 | 10.89 | 6.63 | 3.58 | 2.53 | 1.21 | 0.37 | 0.47 | 0.21 | 0.11 |

*Note.* We compared the ACE frequencies with the ageing sub-sample of the German NAKO public health cohort study (n = 37 890, > 60 years). In the NAKO study about 32% reported one or more ACEs compared to 49% in the present sample. This may be attributed to differences in the questionnaires: the ACE-questionnaire involves a question on “losing a parent through divorce” which was answered positively by 22% in the present sample and was not asked in the NAKO study. Frequencies of emotional, physical, and sexual abuse as well as emotional or physical neglect ranged between 6-15% in the NAKO sample and 3-15% in the present sample (41).

| **Supplementary Table 5.** Non-response analysis comparing individuals who opted in/out for the MRI scan. | | | | |
| --- | --- | --- | --- | --- |
|  | **No MRI** | **MRI** | ***p*** | **SMD^1^** |
| **n** | 7 479 | 2 525 |  |  |
| age (mean (SD)) | 61.81 (8.44) | 64.01 (8.27) | <0.001 | 0.263 |
| sex = females (%) | 4 005 (53.5) | 1 103 (43.8) | <0.001 | 0.197 |
| SES-Index (mean (SD)) | 12.49 (3.36) | 12.79 (3.45) | <0.001 | 0.088 |
| ACE-sum (mean (SD)) | 1.18 (1.64) | 1.07 (1.56) | 0.007 | 0.067 |
| PHQ-9 score | 2.42 (1.96) | 2.26 (1.96) | 0.001 | 0.081 |
| GAD-7 score | 2.21 (1.98) | 2.01 (1.95) | <0.001 | 0.099 |
| *Note.* ^1^SMD = standardized mean difference. | | | | |

**Supplementary Table 6.** Descriptive statistics of the imputed sample (n = 2 042).


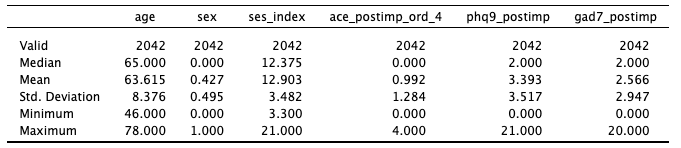


| **Supplementary Table 7.** Results of generalized additive models for assessment of non-monotonic relationships. | | | | |
| --- | --- | --- | --- | --- |
| **Model** | **Smooth term (edf)** | ***p-value***  **(smooth)** | **Age effect**  **(p-value)** | **Sex effect**  **(p-value)** |
| Bilateral amygdala ~  ace score | 1.004 | 0.291 | <2x10^-16^ *** | <2x10^-16^ *** |
| Bilateral hippocampus ~ ace score | 1.001 | 0.777 | <2x10^-16^ *** | <2x10^-16^ *** |
| DLPFC ~ ace score | 2.661 | 0.229 | <2x10^-16^ *** | 1.8x10^-07^ ** |
| PHQ-9 score ~  bilateral hippocampus | 1.625 | 0.294 | 1.21x10^-07^ *** | 2.95x10^-09^ *** |
| PHQ-9 score ~  bilateral amygdala | 1 | 0.199 | 1.28x10^-07^ *** | 2.83x10^-08^ *** |
| PHQ-9 score ~ DLPFC | 1.001 | 0.234 | 7.62x10^-08^ *** | 3.37x10^-12^ *** |
| GAD-7 ~  bilateral hippocampus | 2.044 | 0.379 | 3.82x10^-09^ *** | 4.49x10^-09^ *** |
| GAD-7 ~  bilateral amygdala | 1 | 0.00823 ** | 1.27e-11 *** | 2.96x10^-06^ *** |
| GAD-7 ~ DLPFC | 1.007 | 0.828 | 9.97x10^-10^ *** | 6.06x10^-11^ *** |
| *Note.* Significance codes: <0.001***, <0.01*; the three sensitivity analyses (in (1) the sample excluding outliers, (2) the imputed sample, and (3) the imputed sample excluding outliers) confirmed these results. | | | | |

| **Supplementary Table 8.** Prior Sensitivity Analyses. | | | | | |
| --- | --- | --- | --- | --- | --- |
| **Run** | **Model prior** | **Coefficient priors** | | **BF_10_** | |
|  |  | **Fixed r** | **Covariate r** | **PHQ-9**^1^ | **GAD-7**^1^ |
| **1** | Uniform | 0.5 | 0.354 | \| 3.887×10^+36^ \|  \| \| --- \| --- \| | 3.060×10^+30^ |
| **2** | Uniform | 0.2 | 0.2 | \| 3.389×10^+36^ \|  \| \| --- \| --- \| | \| 2.859×10^+30^ \|  \| \| --- \| --- \| |
| **3** | Uniform | 1.0 | 1.0 | 1.641×10^+36^ | 1.215×10^+30^ |
| **4** | Uniform | 1.5 | 1.5 | 6.279×10^+35^ | 4.253×10^+29^ |
| **5** | Castillo | 0.5 | 0.354 | 3.861×10^+36^ | 3.106×10^+30^ |
| *Note.* BF = Bayes Factor. ^1^Dependent variable of the respective model. | | | | | |

| **Supplementary Table 9.** Post-hoc comparisons of PHQ-9 scores across groups with ACE_0_-ACE_4+_. | | | | | |
| --- | --- | --- | --- | --- | --- |
| **ACE score** |  | **Prior odds** | **Posterior odds** | **BF_10_** | **error %** |
| 0 | 1 | 1.000 | 21.292 | 21.292 | 0.001 |
|  | 2 | 1.000 | 4.370×10^7^ | 4.370×10^7^ | 6.467×10^-10^ |
|  | 3 | 1.000 | 7943×10^13^ | 7.943×10^13^ | 2.250×10^-20^ |
|  | 4+ | 1.000 | 1.611×10^36^ | 1.611×10^36^ | 7.607×10^-43^ |
| 1 | 2 | 1.000 | 24.950 | 24.950 | 9.157x10^-4^ |
|  | 3 | 1.000 | 505130.050 | 505130.050 | 4.496×10^-4^ |
|  | 4+ | 1.000 | 7.741×10^17^ | 7.741×10^17^ | 1.623x10^-24^ |
| 2 | 3 | 1.000 | 1.483 | 1.483 | 0.013 |
|  | 4+ | 1.000 | 51778.470 | 51778.470 | 4.163×10^-11^ |
| 3 | 4+ | 1.000 | 2.529 | 2.529 | 0.008 |
| *Note.* Posterior odds have been corrected for multiple testing. Individual comparisons are based on the default t-test with a Cauchy (0, r = 1/sqrt(2)) prior. The Bayes factor is uncorrected. | | | | | |

| **Supplementary Table 10.** Post-hoc comparisons of GAD-7 scores across groups with ACE_0_-ACE_4+_. | | | | | |
| --- | --- | --- | --- | --- | --- |
| **ACE score** |  | **Prior odds** | **Posterior odds** | **BF_10_** | **error %** |
| 0 | 1 | 1.000 | 35.644 | 35.644 | 6.350x10^-4^ |
|  | 2 | 1.000 | 61395.435 | 61395.435 | 4.212×10^-7^ |
|  | 3 | 1.000 | 4.706x10^16^ | 4.706x10^16^ | 3.995×10^-23^ |
|  | 4+ | 1.000 | 9.217x10^30^ | 9.217x10^30^ | 1.627×10^-37^ |
| 1 | 2 | 1.000 | 0.805 | 0.805 | 0.025 |
|  | 3 | 1.000 | 1.021x10^12^ | 1.021x10^12^ | 2.178×10^-12^ |
|  | 4+ | 1.000 | 5.894×10^12^ | 5.894×10^12^ | 2.818×10^-19^ |
| 2 | 3 | 1.000 | 24.511 | 24.511 | 0.001 |
|  | 4+ | 1.000 | 22554.414 | 22554.414 | 9.809×10^-11^ |
| 3 | 4+ | 1.000 | 0.309 | 0.309 | 0.050 |
| *Note.* Posterior odds have been corrected for multiple testing. Individual comparisons are based on the default t-test with a Cauchy (0, r = 1/sqrt(2)) prior. The Bayes factor is uncorrected. | | | | | |

**Supplementary Table 11.** Results of the sensitivity analysis (ANCOVA) for the dependent variable PHQ-9 score using the complete cases sample excluding outliers (n = 1 815).

*Note.* All models include sex and age. Independent variable: ACE. The mean PHQ-9 sum score was 2.45 (SD = 2.66) for individuals with ACE_0_ compared to 5.30 (SD = 3.71) for individuals with ACE_4+_.


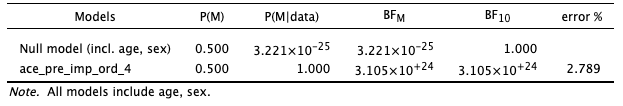


**Supplementary Table 12.** Results of the sensitivity analysis (ANCOVA) for the dependent variable GAD-7 score using the complete cases sample excluding outliers (n = 1 815).

*Note.* All models include sex and age. Independent variable: ACE. The mean GAD-7 sum score was 1.82 (SD = 2.15) for individuals with ACE_0_ compared to 3.85 (SD = 2.92) for individuals with ACE_4+_.


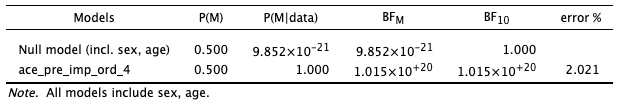

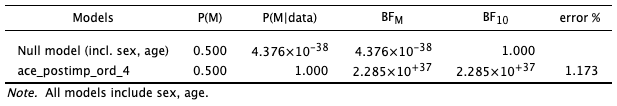


**Supplementary Table 13.** Results of the sensitivity analysis (ANCOVA) for the dependent variable PHQ-9 score using the imputed sample (n = 2 042).

*Note.* All models include sex and age. Independent variable: ACE. The mean PHQ-9 sum score was 2.62 (SD = 2.97) for individuals with ACE_0_ compared to 6.38 (SD = 4.61) for individuals with ACE_4+_.

**
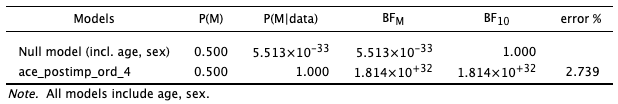
**

**Supplementary Table 14.** Results of the sensitivity analysis (ANCOVA) for the dependent variable GAD-7 score using the imputed sample (n = 2 042).

*Note.* All models include sex and age. Independent variable: ACE. The mean GAD-7 sum score was 1.95 (SD = 2.42) for individuals with ACE_0_ compared to 4.81 (SD = 3.81) for individuals with ACE_4+_


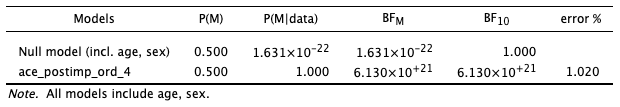


**Supplementary Table 15.** Results of the sensitivity analysis (ANCOVA) for the dependent variable PHQ-9 score using the imputed sample excluding outliers
(n = 1 945).

*Note.* All models include sex and age. Independent variable: ACE. The mean PHQ-9 sum score was 2.43 (SD = 2.56) for individuals with ACE_0_ compared to 5.13 (SD = 3.38) for individuals with ACE_4+_.


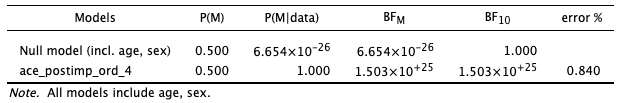


**Supplementary Table 16.** Results of the sensitivity analysis (ANCOVA) for the dependent variable PHQ-9 score using the imputed sample excluding outliers
(n = 1 945).

*Note.* All models include sex and age. Dependent variable: ACE. Independent variable: GAD-7 score. The mean GAD-7 sum score was 1.81 (SD = 2.11) for individuals with ACE_0_ compared to 3.83 (SD = 2.82) for individuals with ACE_4+_

| **Supplementary Table 17.** Significant clusters of reduced rGMV in individuals with ACE_4+_ compared to those with ACE_0_. | | | |
| --- | --- | --- | --- |
| **Cluster size** | **p-level** | **Peak  coordinate** | **Region(s)** |
| 11 036 | 7.7e-08 | 26 40 42 | Left and right frontal gyrus including dorsolateral, medial, and orbital parts  Left and right medial orbital gyrus  Left and right anterior cingulate cortex, pre- and subgenual  Left and right NAcc  Left and right olfactory bulb  Left and right NAcc  Left and right gyrus rectus |
| 1 658 | 6.7e-07 | -44 42 6 | Left Inferior frontal gyrus-triangular part  Left middle frontal gyrus |
| 660 | 3.6e-06 | 58 -14 -12 | Right middle and superior temporal gyrus |
| 437 | 3.1e-06 | -50 -40 50 | Left inferior parietal gyrus |
| 411 | 5.1e-06 | -40 -3 10 | Left Rolandic operculum  Left insula |
| 401 | 7.7e-06 | -21 -70 -45 | Left Lobule VIII of cerebellar hemisphere |
| 264 | 6e-06 | -38 -76 3 | Left middle and inferior occipital gyrus |
| 255 | 5.5e-06 | -62 -62 -3 | Left middle temporal gyrus |
| 253 | 6.3e-06 | 51 -42 -24 | Right Inferior temporal gyrus  Right fusiform gyrus |
| 248 | 5e-06 | -4 21 34 | Left middle cingulate and paracingulate gyri |
| 238 | 3.5e-06 | -52 -32 -10 | Left middle temporal gyrus |
| 229 | 9.4e-06 | 20 -58 -28 | Right Lobule VI of cerebellar hemisphere |
| 200 | 1e-06 | -50 6 44 | Left middle frontal gyrus  Left precentral gyrus |
| 200 | 4.8e-06 | 36 40 -18 | Right anterior orbital gyrus  Right inferior frontal gyrus pars orbitalis  Right middle frontal and lateral orbital gyrus |
| 128 | 1.2e-05 | -28 3 -45 | Left fusiform gyrus |
| 123 | 1.6e-05 | 62 -56 -9 | Right Inferior temporal gyrus |
| 106 | 1e-05 | 48 -82 -6 | Right Inferior occipital gyrus |
| 88 | 6.1e-06 | 39 42 38 | Right middle frontal gyrus |
| 83 | 7.9e-06 | -64 -22 -6 | Left Middle temporal gyrus |
| 77 | 3.8e-06 | 51 14 30 | Right Precentral gyrus |
| 60 | 1.1e-05 | -50 -27 2 | Left superior temporal gyrus |
| 56 | 3.4e-06 | 56 -66 -44 | Left middle temporal gyrus |
| *Note.* The cluster extent threshold was k > 37. The table displays FWE-corrected peak-level p-values. The sample included n = 161 individuals with ACE_4+_ and n = 958 with ACE_0._ | | | |
